# A survey of national and multi-national registries and cohort studies in juvenile idiopathic arthritis: challenges and opportunities

**DOI:** 10.1186/s12969-017-0161-5

**Published:** 2017-04-19

**Authors:** Timothy Beukelman, Janneke Anink, Lillemor Berntson, Ciaran Duffy, Justine A. Ellis, Mia Glerup, Jaime Guzman, Gerd Horneff, Lianne Kearsley-Fleet, Ariane Klein, Jens Klotsche, Bo Magnusson, Kirsten Minden, Jane E. Munro, Martina Niewerth, Ellen Nordal, Nicolino Ruperto, Maria Jose Santos, Laura E. Schanberg, Wendy Thomson, Lisette van Suijlekom-Smit, Nico Wulffraat, Kimme Hyrich

**Affiliations:** 1 University of Alabama at Birmingham, Birmingham, USA; 2 Erasmus Medical Center, Rotterdam, The Netherlands; 3 Uppsala University Hospital, Uppsala, Sweden; 4 Children’s Hospital of Eastern Ontario, Ottawa, Canada; 5 Murdoch Children’s Research Institute, Genes, Environment & Complex Disease, Parkville, Australia; 6 Aarhus University Hospital, Aarhus, Denmark; 7 University of British Columbia, Vancouver, Canada; 8 Asklepios Klinik Sankt Augsutin, Sankt Augustin, Germany; 9 University of Manchester, Manchester, UK; 10 German Rheumatism Research Center, Berlin, Germany; 11 Karolinska University Hospital, Stockholm, Sweden; 12 Royal Children’s Hospital, Melbourne, Australia; 13 University Hospital of North Norway, Tromso, Norway; 14 Istituto Giannina Gaslini, Genoa, Italy; 15 Hospital Garcia de Orta, Almada, Portugal; 16 Duke University, Durham, USA; 17 University Medical Center Utrecht, Utrecht, The Netherlands; 18 University of Manchester and Central Manchester Foundation Trust, Manchester, UK; 19 Division of Pediatric Rheumatology, The University of Alabama at Birmingham, 1600 7th Avenue South, CPP 210, Birmingham, AL 35233-1711 USA

**Keywords:** Juvenile idiopathic arthritis, Registry, Observational study, Pharmacosurveillance, Pediatric rheumatology

## Abstract

**Background:**

To characterize the existing national and multi-national registries and cohort studies in juvenile idiopathic arthritis (JIA) and identify differences as well as areas of potential future collaboration.

**Methods:**

We surveyed investigators from North America, Europe, and Australia about existing JIA cohort studies and registries. We excluded cross-sectional studies. We captured information about study design, duration, location, inclusion criteria, data elements and collection methods.

**Results:**

We received survey results from 18 studies, including 11 national and 7 multi-national studies representing 37 countries in total. Study designs included inception cohorts, prevalent disease cohorts, and new treatment cohorts (several of which contribute to pharmacosurveillance activities). Despite numerous differences, the data elements collected across the studies was quite similar, with most studies collecting at least 5 of the 6 American College of Rheumatology core set variables and the data needed to calculate the 3-variable clinical juvenile disease activity score. Most studies were collecting medication initiation and discontinuation dates and were attempting to capture serious adverse events.

**Conclusion:**

There is a wide-range of large, ongoing JIA registries and cohort studies around the world. Our survey results indicate significant potential for future collaborative work using data from different studies and both combined and comparative analyses.

## Background

Juvenile idiopathic arthritis (JIA), a heterogeneous collection of inflammatory arthritides, is the most common rheumatic condition of childhood [1]. Despite this, our understanding of the long-term outcomes for many children with this condition, in terms of disease status, functional limitation, need for long-term immunosuppression as well as the development of comorbidities, remains relatively limited. Similarly, we have an incomplete understanding of the potential adverse effects of new therapeutic agents, especially effects that are rare or have a long latency period.

The epidemiological study of JIA is challenging. Compared to the 1% prevalence of rheumatoid arthritis (RA) in adults, JIA is rare. The approximate worldwide yearly incidence is 8 per 100,000 children and most prevalence estimates range from 15 to 150 per 100,000 depending on geographic region and study methodology [2]. In contrast to the categorization of the arthritis counterparts in adulthood, JIA encompasses a wide range of inflammatory arthritides, from self-limited oligoarthritis to severe, persistent polyarthritis, and it also includes psoriatic arthritis, spondyloarthritis, and systemic arthritis (an autoinflammatory condition). Results from long-term follow-up studies of JIA may not be broadly generalizable; they may exclude children with milder forms of disease who are discharged from rheumatology care, and they may include too few children to adequately study the less common disease phenotypes. The long-term study of childhood-onset diseases is complicated when patients transition to the adult healthcare setting, making the capture of health outcome data challenging. However, patients and parents identify long-term outcomes, such as physical ability in adulthood, successful schooling and employment, and the consequences of long-term exposure to immunosuppression in childhood, as some of their key priorities for research [3, 4].

With the advent of biological therapies and their increasing use, reliable outcome data in JIA has become even more crucial. Little is known about the background rates of serious outcomes, such as infections and malignancies, making it difficult to interpret the safety of new medications. Extrapolation from studies of these drugs in adults with RA has limited accuracy because most children with JIA do not have RA (only approximately 5% have rheumatoid factor positive polyarthritis) and children have far fewer serious comorbid conditions compared to adults. Exposure to immune modulating drugs in developing immune systems may also result in different longer-term adverse health outcomes compared to more mature immune systems.

Given the rarity of JIA and the now frequent use of potentially high risk treatments in the absence of robust, large, long-term safety studies, the capture of data in longitudinal cohorts is essential. Appropriately combining data from various cohorts enables detection of rare adverse events and more accurate assessment of disease outcomes. Comparing data from different countries may provide information about genotypic variations in disease and geographic variation in environmental risk factors. It may also allow for comparisons of treatment differences that result largely from local availability of therapeutic agents rather than differences in disease characteristics.

The purpose of this study was to survey the international landscape of prospective longitudinal registries and cohort studies in JIA to assess the breadth and depth of data being collected. This information will inform future international collaborative work in JIA, including data harmonization for future studies.

## Methods

A list of existing JIA registries and cohort studies was compiled through known contacts of the authors, ongoing collaborations, and a review of the recent literature. This included studies conducted in the United States of America (USA), Canada, United Kingdom (UK), Germany, the Netherlands, Sweden, Norway, Portugal, and Australia, as well as some multi-national studies. A survey developed in Microsoft Excel (2010) was emailed to the principal investigators of each registry or cohort. The survey also asked for additional registries or cohorts known to the respondent. Surveys were completed between October and December 2014. Owing to delays in manuscript preparation and in an effort to provide the most recent information possible, all respondents were given the opportunity to revise their responses in March 2016.

The survey captured details about each registry or cohort study, including study design, study duration, location, inclusion criteria and number of participants, and data elements and collection method. Cross-sectional studies, defined as those with less than 12 months of planned follow-up per participant, were excluded.

## Results

In total, 20 investigators were contacted, and the replies described 20 studies. Two identified studies were not included (CLARITY from Australia [5] and EPOCA from PRINTO [6]) due to cross-sectional design. The remaining 18 studies contain more than 60,000 patients with JIA (Table 1). These studies include 11 national and 7 multi-national cohorts (covering more than 37 countries in total), with data spanning from 1993 until 2016 onwards. These included 5 JIA inception cohorts (recruiting exclusively from disease onset), 6 prevalent disease cohorts (without strict requirements about disease duration or treatment), and 7 treatment cohorts (restricted to children starting certain anti-rheumatic therapies, such as biologics). Data from treatment cohorts were often part of pharmacosurveillance regulatory requirements. A majority of studies had been established within the past 15 years. Despite the long duration of some studies, the number of total subjects recruited to each study remained relatively low, with few national studies exceeding 1500 children.

**Table 1:** Summary of JIA registries and cohort studies

Study Name and Published Reference	Country	Start Year	Approx. number of patients	Children/adults enrolled	Minimum Follow-up (Years)	Active Follow-up after Transition of Care	Active Recruitment	Active Follow-up
Inception Cohorts								
CAPS [Childhood Arthritis Prospective Study] [19]	UK	2001	1500	Children	10 years or transition	No (patient questionnaire at age 18 and 21 years)	Yes	Yes
ICON [Inception Cohort of Newly Diagnosed Children with JIA] [35]	Germany	2010	950	Children	10	Yes	No	Yes
JACS [Juvenile Arthritis Cohort Study]	Australia	2012	25	Children	To transition	No	Yes	Yes
Nordic JIA Cohort [24]	Sweden, Finland, Denmark, Norway, Iceland	1997	1200	Children	15	Yes	Yes	Yes
ReACCh Out [Research in Arthritis in Canadian Children Emphasizing Outcomes] [36]	Canada	2005	1500	Children	5	No	No	Yes
Prevalent Disease Cohorts								
CARRA Legacy [Childhood Arthritis and Rheumatology Research Alliance] [37]	USA, Canada	2010	6600	Children	Until 2013	No	No	No
CARRA (current)^a^ [Childhood Arthritis and Rheumatology Research Alliance] [14]	USA, Canada	2015	994	Children	10	Yes, via Call centre	Yes	Yes
NPRD [National Pediatric Rheumatology Database] [38]	Germany	1997	~29,000	Children	Unlimited	No	Yes	Yes
PHARMACHILD (Retrospective) [Pharmacovigilance in Juvenile Idiopathic Arthritis Patients]	Multi-national	2011	7000	Children	Retrospective data collection only	No	Yes	Yes
Reuma.pt [9]	Portugal/Brazil	2009	1188/310	Both	Unlimited	Yes	Yes	Yes
Swedish JIA Register	Sweden	2009	1800	Children	To transition	No	Yes	Yes
Treatment Cohorts								
ABC [Arthritis and Biologicals in Children] [39]	Netherlands	1999	425	Children	To transition	No	No	No
BiKeR [Biologics in Pediatric Rheumatology] [10, 11]	Germany, Austria	2001	3743	Children	To transition	No	Yes	Yes
JuMBO [Juvenile Arthritis Methotrexate Biologics Long-Term Observation] [8]	Germany	2007	1100	Adults	10	Follow-up to BIKER study	Yes	Yes
BCRD^b^ [Biologics for Children with Rheumatic Diseases] [13]	UK	2010	850	Children	10	Yes	Yes	Yes
BSPAR-ETN^b^ [British Society for Pediatric and Adolescent Rheumatolgy-Etanercept] [12]	UK	2003	1400	Children	Unlimited	Yes	Yes	Yes
BSRBR^c^ [British Society for Rheumatology Biologics Register] [7]	UK	2001	550	Adults	Unlimited	N/A	Yes	Yes
PHARMACHILD (Prospective) [Pharmacovigilance in Juvenile Idiopathic Arthritis Patients]	28 countries	2011	1450	Children	10	Yes, via JAMAR questionnaire	Yes	Yes

^a^The CARRA Registry preferentially enrols children with systemic arthritis, polyarticular JIA, new diagnosis of JIA, or initiation of treatment with methotrexate or biologic agents

^b^BCRD and BSPAR-ETN preferentially recruit children at point of starting MTX or a biologic drug

^c^BSRBR recruits adults starting a biologic drug. The primary focus is RA but includes adults with JIA

Most studies are still recruiting and continuing to follow-up children previously recruited. The planned length of follow-up varied across studies and was generally dependent on the amount or nature of funding available (data not shown). A majority of studies were based in a single country or a small number of countries within a geographic area. One noted exception is the large multi-national Pharmacovigilance in Juvenile Idiopathic Arthritis Patients (Pharmachild) collaboration,which was initiated with a European Union grant from the Framework Programme 7, managed by the Pediatric Rheumatology International Trials Organization (PRINTO), and has recruited children from 28 countries.

The vast majority of studies recruited children, although 2 studies (The British Society for Rheumatology Biologics Register (BSRBR) [7] and The German Juvenile Arthritis - Methotrexate/Biologics Long-term Observation Study (JUMBO) [8], specifically recruit adult patients with JIA receiving biologic therapies. Most studies stop follow-up after children leave paediatric care; however, there are several exceptions. The Portuguese register, Reuma.pt, is built into routine care and continues to capture data from patients into adulthood [9]. JUMBO is a follow-on study that links adults who were followed in the German BiKeR registry [10, 11] as children. Similarly, the UK BSPAR [12] and BCRD registers [13] continue to capture outcome data about young adults from their adult care providers. The CARRA Registry follows young adults after transition of care via a structured survey conducted by a telephone call center [14]. Pharmachild has the option for adult rheumatology centers to submit data and plans to collect data directly from young adults after transition of care by use of the JAMAR questionnaire [15].

Despite differences in geographic location, time since study inception, and study designs, the data elements collected across studies appears to be quite similar (Table 2). All studies but one capture the International League of Associations for Rheumatology (ILAR) category of JIA [1]. More than two-thirds of studies capture the full American College of Rheumatology JIA core outcome set (count of joints with active arthritis, count of joints with limited range of motion, physician global assessment of overall disease activity, patient/parent global assessment of overall well-being, functional ability (Childhood Health Assessment Questionnaire (CHAQ) score), inflammatory markers) [16]. Nearly all of the remaining studies capture 5 of the ACR core outcome variables. Fifteen studies (83%) collect the data needed to calculate the 10 or 71-joint Juvenile Arthritis Disease Activity Score (JADAS-10 or JADAS-71) [17], and 16 can calculate the 3-variable clinical JADAS (cJADAS) which omits inflammatory markers [18]. All studies enrolling children captured data on the presence or absence of uveitis. Most studies (83%) captured medication start and stop dates. Fourteen studies (78%) captured the occurrence of serious adverse events (e.g., requiring hospitalisation), but only 28% had the ability to capture these data additionally through external data sources (the majority based in the UK). Seven registries were working with industry to perform pharmacosurveillance studies and included monitoring of data and adjudication of adverse event reports.

**Table 2:** Data Capture Across JIA Registries and Cohort Studies

Study	ILAR Category	AJC	LJC	MD global	Parent global	CHAQ/HAQ	ESR	CRP	Full ACR Core Set	JADAS	cJADAS	Pain	Uveitis data	JIA Medication Start/Stop dates	Serious Adverse Events	Stored Bio-samples	Imaging results	Link to external data sources	Industry pharmaco-surveillance studies
Inception Cohorts																			
CAPS	Y	Y	Y	Y	Y	Y	Y	Y	Y	Y	Y	Y	Y	Y	Y	Y	Y	Y	N
ICON	Y	Y	Y	Y	Y	Y	Y	Y	Y	Y	Y	Y	Y	Y	Y	Y	Limited	N	N
JACS	Y	Y	Y	Y	Y	Y	Y	Y	Y	Y	Y	Y	Y	Y	Y	Y	N	Y	N
Nordic JIA Cohort	Y	Y	Y	Y	Y	Y	Y	Y	Y	Y	Y	Y	Y	N	N	Y	N	N	N
ReACCh Out	Y	Y	Y	Y	Y	Y	Y	Y	Y	Y	Y	Y	Y	Y	N	Y	N	N	N
Prevalent Cohorts																			
CARRA Legacy	Y	Y	N	Y	Y	Y	N	N	N	N	Y	Y	Y	N	Limited	N	Y	N	N
CARRA (current)	Y	Y	Y	Y	Y	Y	Y	Y	Y	Y	Y	Y	Y	Y	Y	Limited	Y	Potential	Y
NPRD	Y	Y	Y	Y	Y	Y	Y	Y	Y	Y	Y	Y	Y	Start only	Y	Limited	N	N	N
PHARMACHILD (retrospective)	Y	N	N	N	N	N	N	N	N	N	N	N	Y	Y	Y	N	N	N	N
Reuma.pt	Y	Y	Y	Y	Y	Y	Y	Y	Y	Y	Y	Y	Y	Y	Y	Limited	Limited	N	N
Swedish JIA Register	Y	Y	N	Y	Y	Y	Y	Y	N	Y	Y	Y	Y	Y	N	Limited	N	N	Y
Treatment Cohorts																			
ABC	Y	Y	Y	Y	Y	Y	Y	N	Y	Y	Y	Y	Y	Y	Y	Y	Limited	N	N
BCRD	Y	Y	Y	Y	Y	Y	Y	Y	Y	Y	Y	Y	Y	Y	Y	Y	N	Y	N
BIKER	Y	Y	Y	Y	Y	Y	Y	Y	Y	Y	Y	Y	Y	Y	Y	Y	N	N	Y
BSPAR-ETN	Y	Y	Y	Y	Y	Y	Y	Y	Y	Y	Y	Y	Y	Y	Y	Y	N	Y	Y
PHARMACHILD (prospective)	Y	Y	Y	Y	Y	N	Y	Y	N	Y	Y	Y	Y	Y	Y	Limited	Y	N	Y
BSRBR	N	N^a^	N	N	Y	Y	Y	Y	N	N	N	N	N	Y	Y	Limited	N	Y	Y
JuMBO	Y	Y	Y	Y	Y	Y	Y	Y	Y	Y	Y	Y	Y	Y	Y	Limited	N	N	Y

*ILAR* International League of Associations for Rheumatology, *AJC* active joint count, *LJC* limited joint count, *MD* physician, *(C)HAQ* (Childhood) Health Assessment Questionnaire, *ESR* erythrocyte sedimentation rate, *CRP* C-reactive protein, *ACR* American College of Rheumatology, *JADAS* Juvenile Arthritis Disease Activity Score, *cJADAS* clinical (3-variable) Juvenile Arthritis Disease Activity Score, *Y* yes, *N*-no

^a^Captures 28-swollen and tender joint count only

Biosample collection was performed in most of the registries. Nine registries had relatively systematic collection, and 7 had more limited collection on a subset of patients. Serum, plasma, and whole blood/DNA were the most commonly collected samples. Synovial fluid was collected less frequently.

## Discussion

This is the first report to bring together information about the numerous existing JIA registries and longitudinal cohort studies, highlighting a wealth of JIA outcome data being collected around the world. Many important questions surround the etiology, pathogenesis, optimal management, and long-term outcomes of JIA. No single registry can answer all questions. Registries must have a clearly defined purpose that determines the process of data collection and the specific data items collected.

Three primary patient enrolment strategies are being used: diagnosis inception cohorts, prevalence or convenience cohorts, and treatment initiation cohorts. Although all studies aim to assess disease outcomes over time, inception cohorts better identify predictors of short and long-term outcomes (e.g. social, clinical, psychological, laboratory and genetic) [19], whereas treatment initiation cohorts are superior to evaluate treatment effectiveness and safety. Of course, with sufficient patient enrolment and procedures to collect data at times when medications are newly started, treatment cohort studies can be performed within a subset of patients from any registry.

Whilst one study started collecting data as early as 1993, most initiated recruitment following the introduction of biologic therapies for JIA around 2000. This was likely a direct result of increased monitoring and safety concerns among investigators, sponsors, and patients with JIA. The relatively recent increase of JIA cohort studies coincided with a movement in the late 1990’s to classify children with chronic arthritis into homogenous groups to facilitate research using the ILAR classification [1], and this is reflected by all paediatric studies classifying children according to this system. Most studies continue to actively recruit and follow previously recruited patients.

Unfortunately, many studies cease to follow patients after they transition into adult care. The transition of care period presents many challenges. Patients not only change clinicians and often their place of residence, but also may change medical systems and third-party payers. The most relevant outcome measures may also change, such as a change from active and limited joint counts in paediatric clinics to counts of tender and swollen joints in adult clinics, or change from JADAS to RA disease activity measures (e.g., DAS28).

With the majority of JIA studies initiating in the 2000’s only preliminary results on adult-aged patients are currently available. Two studies specifically assess effectiveness and safety of biologics in adult patients with JIA. The German JuMBO study has published outcome data of 346 children (median age 21 years) who had received the biologic etanercept in childhood [8]. The UK BSRBR study has published data on outcomes of 225 adults with JIA who started a biological therapy in childhood [7]. In previous studies, outcomes of adults with JIA have been assessed cross-sectionally [20–22], retrospectively [23], or by identifying adult patients diagnosed as children from medical records [24–27]. These studies reported approximately 40 to 60% of children with JIA continue to have some level of active disease in adulthood [22, 24, 25, 27–29]. Given the relative rarity of JIA and that not all children continue to have active disease in adulthood, it will be important for registries to share their experiences to develop robust methods to track children through adulthood. Because few patients enrolled in the current studies have reached adulthood, this may be an opportunity now to align the data to be collected in the future across studies.

The studies included in our survey do not reflect the entirety of long-term outcomes studies in JIA. Several recent studies have used administrative data to investigate the risk of rare outcomes in JIA, such as malignancy or serious infection [30–32]. Administrative data sources provide readily available data and often very large sample sizes, but provide scant clinical data compared to clinical cohorts. Accordingly, administrative data are typically most useful for studies of adverse effects of medications, but are less useful for studies of medication effectiveness or long-term outcomes in JIA.

Although there were many differences among the studies, there was significant overlap in the data being collected. This may allow for the determination of a minimal data set that all existing and future prospective observational studies could collect, enabling nearly identical analysis plans to be conducted across all data sources.

Our survey was able to summarize the extent of data items captured, but did not collect estimates of the amount of missing data for each variable. Missing data are a common problem in observational research due to clinic non-attendance by patients, variable clinical need for investigation (e.g. inflammatory marker and other blood testing), and failure to report adverse events in busy outpatient clinics, among others. This can result in biases that require careful consideration of how to handle missing data and missing patients. This issue is compounded further in JIA due to the heterogeneity of disease, with many children who are well being discharged from secondary care and therefore no longer contributing to longer term outcome studies. Also, our survey did not enquire in detail about the timing of data capture, particularly in relation to disease onset or start of therapies.

In order to understand the risk of rare outcomes, large sample sizes and long term follow up are needed. Combining data from multiple studies increases statistical power, but must be done carefully. It is reassuring that there is significant commonality on the data items captured across studies, which may permit the creation of a common data model to pool data. However, differing methods of outcome ascertainment (e.g., assessing adverse events by patient report, physician report, or through an external data source) may produce important differences in results. It will also be important to ensure that no patients are double-counted if they have been simultaneously enrolled in more than one registry effort; this is likely currently best addressed at the individual clinical site level, but assignment of a universal identification number allowing patients to contribute to various different registries over time (e.g., in response to changes in age or geographic location) would have potential additional benefits.

This survey did not account for the differences in health care systems, clinical expertise in JIA, or funding for arthritis medications around the world. Because patients are enrolled from pediatric rheumatology centers, differences in health care systems may influence enrolment into JIA registries through physician referral bias. Differential access to biologics likely results in substantial differences in disease duration and severity at the time of starting treatment. Given the differences in patient enrolment and data collection techniques, as well as geographical differences in treatment approaches, genetic background of the patients, and varying incidences of comorbid conditions and endemic pathogens (such as tuberculosis), simple pooling of data may not be the most appropriate approach to evaluate drug safety or effectiveness. In addition, there may be restrictions on the sharing of data, depending on the ethical approval and patient consent obtained in each study. Overcoming these hurdles to facilitate collaborative research in JIA will be challenging and will require an international effort involving multiple stakeholders. Within the JIA research community, initial steps have been taken to begin to address these obstacles [33].

Sophisticated methods for combining data should be explored, such as nested case-control studies or meta-analyses of individual register data analysis. These approaches were adopted by the EULAR Registers and Observational Drug Studies (RODS) Working Group to explore the risk of malignant melanoma associated with TNFi therapies in patients with RA across Europe [34]. Methodological approaches that consider the individual data source will likely provide more accurate results and new insights about confounders and effect modifiers of outcome. Despite the significant resources required, these analyses would be a unique opportunity to answer critical questions about JIA and should be undertaken.

## Conclusions

This study identified a wide range of ongoing JIA cohort studies and registries around the world. Many of the challenges in the long-term study of childhood-onset diseases were highlighted. Nevertheless, the results indicate significant potential for future collaborative work using both combined and comparative analyses of data from different studies. New approaches to maximise data capture beyond childhood will be crucial to answer important questions about long-term outcomes.

## Abbreviations

ABC: Arthritis and biologicals in children
ACR: American college of rheumatology
AJC: Active joint count
BCRD: Biologics for children with rheumatic diseases
BiKeR: Biologics in pediatric rheumatology
BSPAR-ETN: British society for pediatric and adolescent rheumatolgy-etanercept
BSRBRc: British society for rheumatology biologics register
CAPS: Childhood arthritis prospective study
CARRA: Childhood arthritis and rheumatology research alliance
CHAQ: Childhood health assessment questionnaire
CRP: C-reactive protein
ESR: Erythrocyte sedimentation rate
HAQ: Health assessment questionnaire
ICON: Inception cohort of newly diagnosed children with jia
ILAR: International league of associations for rheumatology
JACS: Juvenile arthritis cohort study
JADAS: Juvenile arthritis disease activity score
JAMAR: Juvenile arthritis multidimensional assessment report
JIA: Juvenile idiopathic arthritis
JuMBO: Juvenile arthritis methotrexate biologics long-term observation
LJC: Limited joint count
MD: Physician
N: No
NPRD: National pediatric rheumatology database
Pharmachild: Pharmacovigilance in juvenile idiopathic arthritis patients
PRINTO: Pediatric rheumatology international trials organization
RA: Rheumatoid arthritis
ReACCh Out: Research in arthritis in canadian children emphasizing outcomes
RODS: Registers and observational drug studies
UK: United Kingdom
USA: United States of America
Y: Yes
